# YIF1B Mutational Dysregulation Drives Cutaneous Melanoma Progression by Remodeling the TME

**DOI:** 10.1155/humu/8907583

**Published:** 2026-05-30

**Authors:** Xiaohan Wang, Yangxu Ding, Wenbo Cai, Chaowei Liu, Huijuan Shi

**Affiliations:** ^1^ Department of Dermatovenereology, The General Hospital of Ningxia Medical University (The First Clinical Medical College of Ningxia Medical University), Yinchuan, China

**Keywords:** CD8^+^ T cell, cutaneous melanoma, somatic mutation, tumor microenvironment, YIF1B

## Abstract

YIF1B, a transmembrane protein involved in intracellular trafficking and signaling, is dysregulated in multiple cancers, but its role in SKCM remains unclear. We integrated multiomics data from cutaneous melanoma cohorts, including gene expression, DNA methylation, and somatic mutation profiles. Using the similarity network fusion (SNF) algorithm, we performed molecular subtyping based on prognosis‐associated epigenetic and mutational features. Finally, the biological role of YIF1B was validated in A‐375 and A‐875 melanoma cell lines using qRT‐PCR, wound healing, and transwell migration/invasion assays following shRNA‐mediated knockdown. Patients assigned to the worst‐prognosis CS5 subtype exhibited poorer survival (*p* < 0.05), characterized by mutation and high YIF1B expression. Mechanistically, we showed that YIF1B impairs tumor‐infiltrating CD8^+^ T cell function. High YIF1B coupled with activation of the IL‐6/JAK pathway produces a positive feedback loop in which tumor proliferation/metastasis is enhanced, as is T cell immunosuppression, eventually culminating in profound immunosuppression and tumor growth. Knockdown of YIF1B by shRNA reduced YIF1B levels in A‐375 and A‐875 cells, leading to suppression of cell motility and proliferation (*p* < 0.05). Whole‐genome sequencing also revealed that activating alterations in IL‐6/JAK pathway genes occur frequently in aggressive cutaneous melanoma and correlate with accelerated progression. We demonstrate that YIF1B drives cutaneous melanoma progression and enhances melanoma cell invasiveness. This function may be further enhanced in combination with activation of the IL‐6/JAK pathway, suggesting an important interaction of signaling processes with immunological selection pressures. Together, YIF1B expression and IL‐6/JAK pathway activation status are potential markers for SKCM prognostication and treatment guidance.

## 1. Introduction

Skin cutaneous melanoma (SKCM) is a highly biologically heterogeneous disease with aggressive progression. The tumor microenvironment (TME) of SKCM acts as a complex defense mechanism that actively suppresses the immune response, greatly reducing the efficacy of immunotherapies [1–5]. Cytotoxic CD8^+^ T lymphocytes, which are primarily responsible for killing tumor cells, progressively lose their proliferative and cytotoxic capacity upon chronic antigen exposure and various TME‐imposed stresses [6–10]. This dysfunction is associated with upregulation of programmed death (PD)‐1 and Tim‐3, leading to T cell exhaustion and eventual failure of global immunosurveillance. The main drivers of T cell exhaustion stem from multiple TME elements, including immunosuppressive cellular compartments, metabolic waste products, hypoxia, and altered signal transduction pathways [11–15].

Recent studies have underscored the pivotal role of the IL‐6 cascade in creating a tumor‐favorable microenvironment [16]. Importantly, the IL‐6/JAK signaling pathway has emerged as a key mediator in SKCM, where persistent activation of this axis promotes tumor proliferation, survival, migration, and metastasis while simultaneously inducing profound immunosuppression. Mechanistically, sustained IL‐6/JAK signaling contributes to CD8^+^ T cell exhaustion by upregulating immune checkpoints, inhibiting the release of cytotoxic factors, and driving an exhaustion‐associated gene signature [17–19]. Although Wnt signaling has also been implicated in SKCM pathogenesis [17, 18], converging evidence suggests that the IL‐6/JAK pathway is more directly linked to T cell dysfunction and poor prognosis in this malignancy.

Serotonin is an endogenous indoleamine widely distributed in mammalian organisms that regulates the production of several cytokines, including IL‐6, TNF‐*α*, and IL‐2 [20]. Pharmacological studies have demonstrated the role of this system in autoimmunity and cancer, modulating processes such as cell differentiation and tumor cell migration/invasion [21]. YIF1B, a protein that binds to the serotonin transporter, is primarily involved in transporting intracellular vesicles from the ER to the Golgi apparatus. YIF1B is a potent oncogene in several cancers, including urothelial carcinoma, colorectal adenocarcinoma, and SKCM, where its expression level serves as a poor prognostic marker [22–26]. Although the involvement of the serotonergic system in carcinogenesis is established, the precise role of YIF1B during tumorigenesis and progression remains largely unknown.

YIF1B is an important regulator of intracellular trafficking and may influence the proper trafficking, localization, and activity of other transmembrane molecules, including cytokine receptors such as IL‐6R. These effects may impact key pathways critical for TME function, thereby determining the course of tumor development. We hypothesize that dysregulated YIF1B expression represents a core biological mechanism that leads to enhanced activation of the IL‐6/JAK signaling pathway within SKCM. This, in turn, drives functional exhaustion of CD8^+^ T lymphocytes and facilitates escape from immunosurveillance.

In conclusion, further studies examining how altered YIF1B expression affects the IL‐6/JAK signal transduction pathway will help to elucidate the mechanisms of immune evasion within the heterogeneous SKCM microenvironment. This may reveal new immune regulatory pathways and provide a basis for developing effective strategies to overcome immunotherapy resistance in SKCM patients. Targeting the YIF1B/IL‐6 axis alongside immune checkpoint inhibitors could offer novel opportunities to rejuvenate exhausted CD8^+^ T cells and establish combinatorial therapeutic strategies that circumvent existing treatment barriers for this challenging cancer.

## 2. Method

### 2.1. Data Preprocessing for SKCM Multiomics and Multicenter Cohorts

We first downloaded the different omics datasets on SKCM, available in TCGA (https://http://portal.gdc.cancer.gov/), including all patients who had complete transcriptomic information, mutation profiles, and related clinical information. TCGAbiolinks was used to retrieve gene expression data as well as mutation data, which was then processed with the maftools package. We applied the SKCM data of nine other research teams to our datasets. We used the limma package for preprocessing (background correction, log‐transformation, and normalizing with a quantile method).

The scRNA‐seq data of SKCM were downloaded from the GEO database with ID GSE179373 and preprocessed by the Seurat package, where the combined and single sample expression matrices were converted to Seurat objects. QC steps, such as removing cells that had less than 200 or more than 2500 detected genes and removing cells whose mitochondrial genes made up more than 10% of their RNA content, were performed. We removed batch effects to correct for technical variation across samples. Principal component analysis (PCA) is used on scaled data for dimension reduction, where we choose to use the first 10 principal components as input for further cluster analysis. The resulting cell clusters are then visualized in two dimensions using the uniform manifold approximation and projection (UMAP) visualization.

### 2.2. Cell Cluster Detection

We used unsupervised clustering methods (FindClusters and FindNeighbors) for cluster detection. A clear separation between groups was obtained using a resolution value of 0.5. Subsequently, cell type annotation of each cluster was performed in an automatic way using marker gene expression with the package called Single R. We then isolated the macrophage population and ran a PCA on it in order to identify macrophage subpopulations.

### 2.3. Multiomics Consensus Integration Analysis

Prior to integration, all samples from the five molecular datasets were matched, and gene expression levels (TPM) were log_2_‐transformed. A gene was considered mutated if it harbored any protein‐altering variant, including frameshift indels, in‐frame insertions/deletions, nonsense/missense mutations, splice‐site alterations, or translation initiation site changes. Genomic features were selected using the getElites function of the MOVICS package [9]. For mRNA data, we first retained the Top 1500 genes with the highest median absolute deviation (method = “mad,” top = 1500). Among these, we further identified prognostic genes using univariate Cox regression based on overall survival, retaining those with *p* < 0.05. For mutational profiles, we initially filtered the Top 5000 most frequently mutated genes via the oncoPrint function (using maftools) and then applied frequency analysis within getElites to select the top 5% most commonly mutated genes (i.e., genes mutated in the highest 5% of the cohort). The selected features from each omics layer were integrated for downstream analysis.

Clustering was performed using 10 algorithms implemented in MOVICS via the methods list option: CIMLR, ConsensusClustering, similarity network fusion (SNF), iClusterBayes, PINSPlus, moCluster, NEMO, IntNMF, COCA, and LRA. All algorithms were run with their respective default parameters as defined in MOVICS v1.0. For SNF, which relies on the SNFtool package, we explicitly set *K* = 20 neighbors, *a* *l* *p* *h* *a* = 0.5, and *T* = 20 iterations for network fusion; Euclidean distance was used to construct the initial similarity matrices. To obtain a robust consensus clustering, we applied getConsensusMOIC with Euclidean distance and average linkage as the clustering method, integrating the results from all 10 algorithms.

### 2.4. qRT‐PCR

Total RNA was extracted from cell samples using 1000 *μ*L of TRIzol reagent. The mRNA expression levels were quantified via primer‐specific amplification using SYBR Green I chemistry, with GAPDH serving as the internal reference gene. The relative gene expression was calculated using the 2^−*Δ* *Δ* *C* *t*^ method. The primer sequences used in this study were as follows: YIF1B—forward: 5 ^′^‐ATGATTGGCGGCGTCCTCAC‐3 ^′^; YIF1B—reverse: 5 ^′^‐AGGTGGAAGGTGAGCCAGTA‐3 ^′^; siRNA 5 ^′^‐GCA GAA UGA UGA ACG CUA ATT‐3 ^′^.

### 2.5. Western Blot

After protein quantification, samples were mixed with loading buffer and deionized water, denatured at 95°C for 10 min, subjected to SDS‐PAGE, and transferred to PVDF membranes. Membranes were blocked with 5% skimmed milk for 2 h, incubated with primary antibodies at 4°C overnight, washed with TBST, incubated with secondary antibodies at room temperature for 1 h, and visualized by chemiluminescence after rewashing.

### 2.6. Migration Assays

Cell invasion was assessed using 24‐well migration and invasion inserts (Corning, United States). The sh‐NC and sh‐YIF1B transfected A‐375 and A‐875 cells were harvested and resuspended in DMEM supplemented with 0.5% fetal bovine serum (FBS). Subsequently, 200 *μ*L of the cell suspension containing 8 × 10^4^ cells was seeded into the upper chamber (8 *μ*m pore size), while 600 *μ*L of DMEM containing 10% FBS was added to the lower chamber as a chemoattractant.

After incubation for 48 h, the inserts were fixed with methanol. The noninvaded cells on the upper surface of the membrane were gently removed with cotton swabs, and the invaded cells on the lower surface were stained with 0.5% crystal violet. Images were captured under a microscope at 20× magnification, and the stained cells were counted. All experiments were performed in triplicate.

### 2.7. Cell Scratch

A‐375 and A‐875 cells were divided into the following groups: NC, sh‐NC, and sh‐YIF1B. Cells in the logarithmic growth phase were seeded into six‐well plates at a density of 4 × 10^5^ cells/well. When the cell confluence reached 90%–95%, a straight scratch was created across the center of each well using a sterile 200 *μ*L pipette tip. The detached cells were gently washed away with phosphate‐buffered saline (PBS), and images were captured immediately to record the initial wound width (0 h). Subsequently, the cells were cultured in serum‐free medium. Images were captured again at 24 and 48 h postscratch. The cell migration area was quantified using ImageJ software. The percentage of cell migration area was calculated using the following formula:

Migration area %=Initial scratch area−Scratch area at 24 h /Initial scratch area×100%.

### 2.8. Cell Culture and Transfection

A‐375 and A‐875 cells were both cultured in RPMI and DMEM media containing 10% FBS, while normal human melanocytes (HEMa‐LP) were cultured in special melanocyte medium. All cell lines were incubated in a constant‐temperature incubator at 37°C with 5% CO_2_. Logarithmic phase cells were selected, and when the cell confluency reached 40%–50%, siRNAs targeting YIF1B were transfected into A‐375 and SK‐MEL‐28A‐875 cells, respectively, using Lipofectamine 2000. Cells were collected 48 h after transfection to extract total protein.

### 2.9. Subcutaneous Tumor Formation Experiment in Mice

Male C57BL/6 mice aged 4–6 weeks were housed in a specific pathogen‐free (SPF) environment. Stable B16‐F10 cells of the sh‐YIF1B and sh‐NC groups were washed three times with precooled PBS and then adjusted to a density of 1 × 10^7^ cells/mL with complete DMEM medium. The cells were gently mixed with precooled Matrigel at a volume ratio of 1:1 on ice. A total of 100 *μ*L of the cell‐Matrigel mixture was subcutaneously injected into the right axilla of each mouse, with five mice in each group, which were labeled separately. Animal experiments were approved by the Animal Ethics and Welfare Committee of Ningxia Medical University (IACUC‐2025009).

### 2.10. Statistical Analysis

All data processing and statistical analysis were conducted using R software (Version 3.6.1) and GraphPad Prism. Student′s *t*‐test and one‐way analysis of variance (ANOVA) were used to determine differences between groups, and a *p* *v* *a* *l* *u* *e* < 0.05 indicated statistical significance.

## 3. Results

### 3.1. Classification of Melanoma Subtypes Based on Multiomics Features

**Figure 1:**
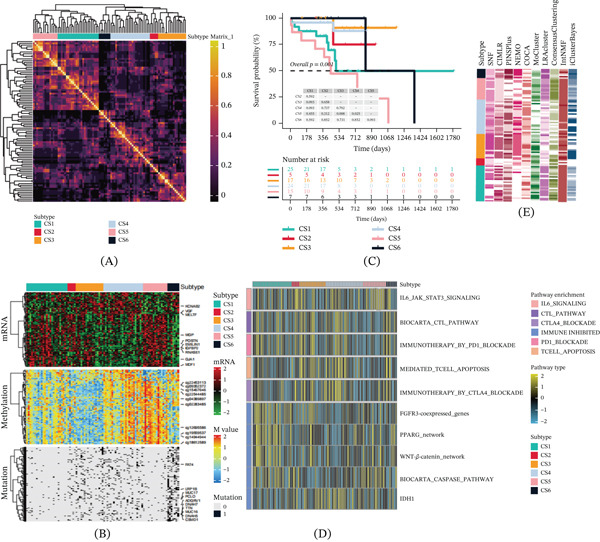
Based on multiomics subgroup identification. (A, B) Heatmap of the gene matrix for SKCM, displaying differential expression, prognosis‐associated methylation sites, and the Top 50 somatic mutation sites. (C) Six SKCM subtypes. (D) Enrichment analysis of subtype pathways.(E) Patient clustering analysis integrated from 10 multiomics clustering algorithms.

Currently, the classification of SKCM mainly depends on molecular expression patterns and clinical–pathological features. According to previous researches, in this study, we propose a novel classification model of SKCM to cope with the high biological variability, particularly for immune and stromal microenvironments as well as genomic stability and tumorigenicity. To better understand the disease mechanisms of cutaneous melanoma, we established a comprehensive data matrix in TCGA‐SKCM patients, combining gene expression data, prognosis‐related DNA methylations, and the most commonly altered sites in genes (Figure 1A,B). We used the CPI algorithm and missing value imputation for estimating an optimal number of clusters. We decided to use *k* = 6 for subsequent analysis. We performed the ssGSEA and classified SKCM into six clusters (CS1–6). We further validated the stability of our classification by running a consensus cluster with 10 algorithms.

The best partitioning (*k* = 6) divided the patients from TCGA‐SKCM into six clusters with different biological characteristics (Figure 1E). The overall survival of each cluster was significantly different among those six clusters (log‐rank *p* *v* *a* *l* *u* *e* < 0.001), wherein patients in CS5 demonstrated the worst outcome and patients in CS3 had the best outcomes (Figure 1C). Importantly, pathway enrichment analysis showed that CS5 was most highly enriched for high activity in multiple important pathways, which include the immune suppression pathway, PD‐1 therapy resistance pathway, and T‐cell exhaustion‐related apoptosis pathway, which indicates that the IL‐6 signal may play a significant role in the progression of melanoma.

### 3.2. Identification of Signaling Pathways, Immune Landscape Differentiation, and CS1 Upregulated Genes Among Subtypes

**Figure 2:**
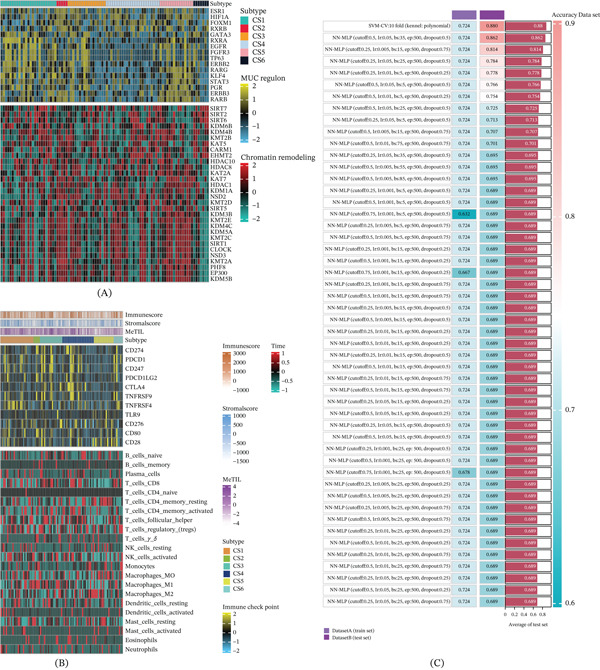
Characteristic analysis of CS subgroups. (A) Chromatin remodeling factors of SKCM subtypes. (B) Analysis of the immune characteristics of the SKCM subpopulation. (C) CS5 subgroup diagnosis based on machine learning models.

In addition, we found that EGFR, RARG, PGR, ERBB3, STAT3, and FGFR3 were significantly overexpressed in the CS5 as well as the CS1 subgroups. We believe these changes in expression levels of factors involved with chromatin remodeling indicate a large difference in regulation for each subgroup, suggesting that the epigenetic regulation of gene regulatory networks could be key determinants in distinguishing between such molecular subtypes (Figure 2A).

Given that the tumor immune microenvironment plays an important role in tumorigenesis and progression, we also compared the infiltrated immune cells between different subtypes to see whether there was any difference. We found lower content of stroma in the CS6 subtype; high heterogeneity was detected for both the expression of immune checkpoint molecules and subsets of immune cells. The least active immune profiles were observed for CS6, while immune activation was higher in CS1–5 (Figure 2B). Given that we previously demonstrated that CS5 is related to the worst clinical outcome of SKCM, we restricted the following analysis to that subpopulation.

We then selected the upregulated genes in CS5 to test whether they can be used as markers for the diagnosis of the disease. We performed extensive simulations using 10 classification algorithms and a total of 101 tuning parameters. We conducted thorough sensitivity and specificity analyses, which verified a good diagnostic value for each marker, especially for the gene YIF1B, which we find to be a predictor of survival (Figure 2C).

### 3.3. YIF1B as a Prognostic Biomarker and Immune Modulator in Cutaneous Melanoma

**Figure 3:**
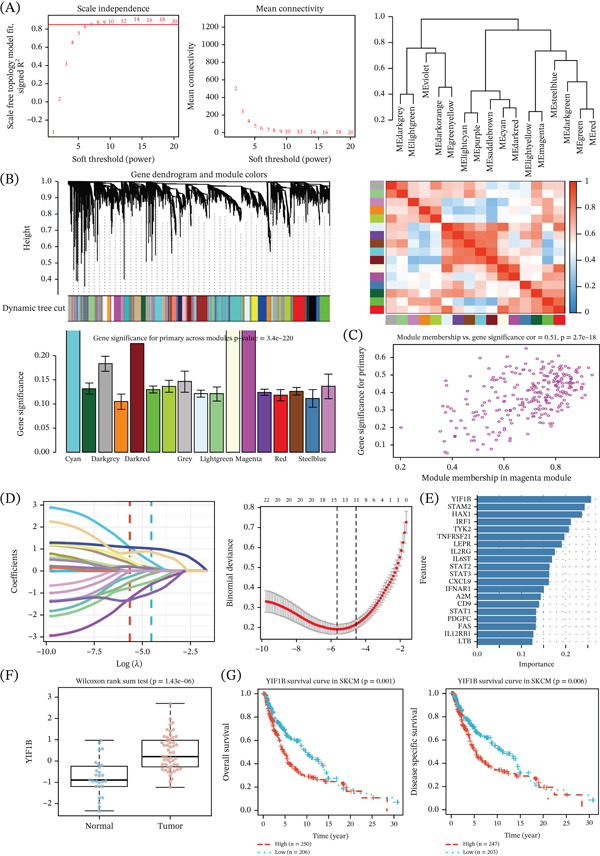
Construction of the hub genes related to the development of cutaneous melanoma. (A, B) WGCNA was used to select modules correlated with diseases. (C) Correlation between the selected modules and traits for melanoma cases was analyzed. (D) LASSO and support vector machine algorithms were used to select genes. (E) The combination of WGCNA and a machine learning model can be used to predict the expression pattern and prognosis‐related gene (YIF1B) (F‐G) in the SKCM dataset.

We identified the core genes for each of the CS subgroups in SKCM. We applied ML and performed a series of screens on CS5‐related genes, and then we validated this result by using the GSE98394 dataset. WGCNA was used to develop a scale‐free neural network model. The best value of the soft‐thresholding parameter is set at 7, where we obtained an optimal network topology with minimal loss of mean connectivity. The correlation among all network modules was strong (Figure 3A). Module eigengenes showed that one of the modules (magtan) has the largest variation between the melanoma samples, suggesting that it may play a role during the progression of the disease (Figure 3B,C).

To determine which are the best candidate genes for this module, we ran LASSO regression with respect to the intersection of genes in this module and CS5 genes (Figure 3D), yielding a total of 20 genes that were found to be shared (Figure 3E). Inspection revealed close connections between each of these module eigengenes and clinically meaningful traits in melanoma; among these, the most interesting was for the gene YIF1B. By evaluating the survival and expression data from the GEO database, we found that compared with normal tissue, a high level of YIF1B was expressed in melanoma specimens. Moreover, higher levels of YIF1B were significantly associated with poor OS and DSS (Figure 3F,G), suggesting that it could be used as a potential biomarker for melanoma diagnosis.

We further analyzed some of the most significantly over‐represented pathways with an absolute value of log‐fold change greater than 1 and a *p* value less than 0.05. We performed Cox proportional hazard analysis on these pathways to find that we identified several paths that are associated with a high risk for melanoma progression. The top hit pathways found included the following: KRAS_DN_4, XENOBIOTIC_METABOLISM_1, UNFOLDED_PROTEIN_RESPONSE_3, NOTCH_SIGNALING_1, INTERFERON_GAMMA_RESPONSE_2, GLYCOLYSIS_4, and especially the IL6_JAK_STAT3_SIGNALING pathway (Supporting Information 1: Figure S1A). The latter showed the highest enrichment out of all found pathways (Supporting Information 1: Figure S1B). In addition, using correlation analyses, we found that the IL6_JAK_STAT3_SIGNALING was the most significantly associated with cutaneous melanoma disease, which implies a potential involvement of the gene during progression of the disease, and for survival prediction, we observed increasing HRs across combined panels, where the IL6_JAK_STAT3_SIGNALING pathway is always included in such prognosis models. Finally, we analyzed in depth all those genes related to this pathway to present data in the form of a volcano plot, and this graphically confirmed a high expression level for gene YIF1B. Furthermore, we also identified that the melanoma is associated with changes in phosphorylation related to oxidative stress through analysis of the metabolism network and activation of the mTOR pathway signaling. This suggests that induction of these pathways is important for the progression of melanomas arising from the skin (Supporting Information 1: Figure S1C).

To further explore whether there are changes to the tumor immune microenvironment (TME) in CM, we also analyzed the immune infiltration profile for genes with significant associations with overall survival (YIF1B). The analysis showed that high levels of YIF1B were significantly correlated with higher infiltration of CD8^+^, Th1 helper cells, and the M0 and M1 macrophage subpopulations (Supporting Information 2: Figure S2A). Furthermore, the high‐risk group patients showed a consistent trend of immune exhaustion correlated to YIF1B expressions (Supporting Information 2: Figure S2B). We divided the level of YIF1B expression into four groups (Group 1: lowest quarter; Group 2: second to third quarters; Group 3: fourth to seventh quarters; Group 4: highest quarter) in order to test for immune checkpoints. This analysis showed that increased expression of YIF1B strongly activated CD80 and CD28 checkpoints, which also significantly affected BTLA, an important extracellular signal transduction receptor (Supporting Information 2: Figure S2C).

### 3.4. Multiomics Revealed That Depletion of CD8T Cells Is Mediated by IL6 Signaling Pathway

**Figure 4:**
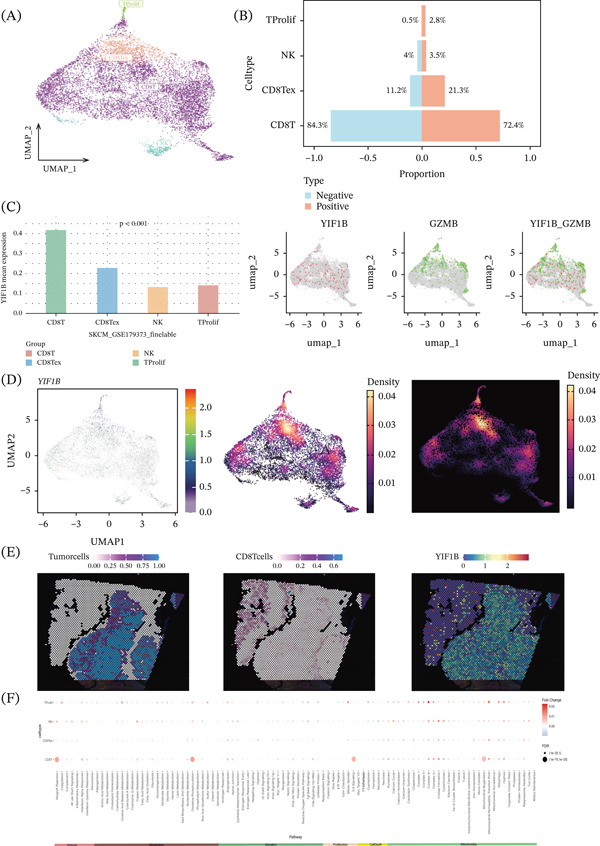
Spatial transcriptome reveals heterogeneity of YIF1B expression in cutaneous melanoma. (A) UMAP shows all cell types. (B) It shows the proportion distribution of different cell types in the YIF1B expression positive and negative groups. In the legend, blue represents the YIF1B negative expression group, and red represents the YIF1B positive expression group. (C) Left: proportion analysis of single‐cell subsets; right: colocalization analysis of YIF1B and GZMB. (D) UMAP expression localization of YIF1B. (E) Spatial expression localization of tumor cells, CD8T cells, and YIF1B. (F) KEGG enrichment analysis of single‐cell subpopulations.

Additional spatiotemporal analyses were performed on a single‐cell level for the data of SKCM_GSE179373, which consisted of four main cell clusters, including terminally differentiated CD8T cells (CD8Tex), natural killer cells, and actively dividing T lymphocytes (Tprolif) [11–13]. The YIF1B protein was enriched in the CD8T lymphocyte compartment (Figure 4A,B). CD8T cell signatures using known CD8T cell signature genes (GZMB), we identified spatial coexpression signatures, including a signature for YIF1B that is enriched among classical CD8T cells, while the highest levels of GZMB were seen in exhausted CD8T cells (Figure 4C). Analysis by UMAP indicated that low levels of YIF1B were expressed across almost every population (Figure 4D). To further explore the possible mechanism, we examined other types of intercellular communications and found that communication mediated by IL‐6 was correlated with poor performance of CD8T cells (Figure 4F). To confirm this finding, we performed spatial transcriptomic analysis on SKCM tumors and found high expression of YIF1B at the center region (regions with reduced T‐cell density and spatial correlations associated with compromised T‐cell activity) (Figure 4E).

### 3.5. Somatic Mutations Drive Oncogenesis in Cutaneous Melanoma

**Figure 5:**
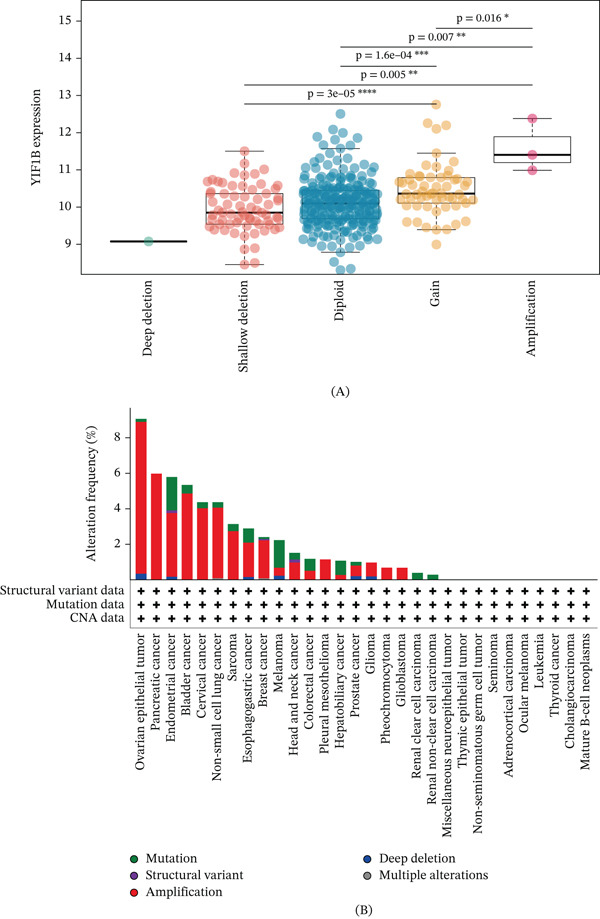
Genomic alterations and expression profiles of YIF1B across pan‐cancer cohorts. (A) Association between YIF1B mRNA expression levels and copy number variations (CNVs). The box plots illustrate YIF1B expression (log_2_‐transformed) across five distinct CNV states: deep deletion, shallow deletion, diploid, gain, and amplification. Statistical significance was determined using [insert test name, e.g., Student′s*t*‐test or ANOVA], indicated by asterisks ( ^∗^ *p* < 0.05,  ^∗∗^ *p* < 0.01,  ^∗∗∗^ *p* < 0.001,  ^∗∗∗∗^ *p* < 0.0001). (B) Landscape of YIF1B genomic alterations across different tumor types. The bar plot displays the frequency of alterations, categorized into amplification (red), mutation (green), and structural variant (dark green/blue). The lower panel indicates the presence of specific alteration types for each cancer cohort.

To investigate the regulation of YYF1B expression by genomic copy number, we analyzed the correlation between YIF1B mRNA levels and CNV status. As shown in Figure 5A, YIF1B expression exhibited a strong positive correlation with copy number status. Samples with YIF1B amplification showed significantly higher expression levels compared to the diploid group, while deep deletions were associated with markedly reduced expression. This suggests a gene dosage‐dependent effect on YIF1B transcription. Furthermore, we characterized the frequency of YIF1B alterations across multiple cancer types (Figure 5B). The analysis revealed that amplification is the predominant type of alteration in several malignancies, particularly in ovarian epithelial tumor, where alteration frequencies exceeded 8%. Mutations and structural variants were observed at lower frequencies across the cohorts. These findings highlight that YIF1B is frequently amplified in specific cancers, potentially driving its overexpression.

### 3.6. Downregulation of YIF1B Expression Inhibits Tumor Proliferation

**Figure 6:**
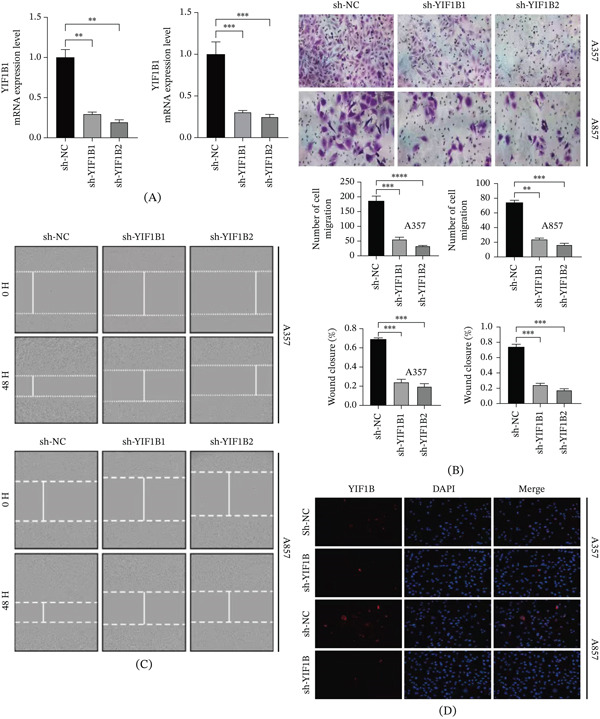
Inhibition of YIF1B expression reduced the progression of cutaneous melanoma. (A) Detection of YIF1B mRNA expression in A‐357 and A‐857 cell lines after transfection with sh‐YIF1B by qPCR. (B, C) Detection of A‐357 and A‐857 cells by transwell and cell scratch assay. Tumor invasion after lines transfected with sh‐YIF1B. (D) Immunofluorescence was used to detect the expression of YIF1B in A‐357 and A‐857 cell lines transfected with sh‐YIF1B  ^∗∗^ *p* < 0.01,  ^∗∗∗^ *p* < 0.001,  ^∗∗∗∗^ *p* < 0.0001.

To test whether high expression of YIF1B contributes to the progression of melanoma, we carried out experiments using A‐357 and A‐857 cells. YIF1B expression was reduced significantly by the shRNA compared with the nontargeting control (Figure 6A). To confirm this result, we performed wound healing and transwell migration assays. The results showed silencing YIF1B significantly inhibited cell migration and proliferation in these two cell lines (Figure 6B,C). And western blot confirmed the efficiency of sh‐YIF1B on downregulating YIF1B expression (Supporting Information 3: Figure S3B). Additionally, we observed that sh‐YIF1B inhibits tumor proliferation (Supporting Information 3: Figure S3A).

## 4. Discussion

In the present study, we identified YIF1B as a key oncogenic driver in SKCM that not only promotes tumor cell proliferation, migration, and invasion but also remodels the TME to facilitate immune escape. Our multiomics and experimental data demonstrate that YIF1B overexpression correlates with poor prognosis and that its protumorigenic effects are closely linked to activation of the IL‐6/JAK signaling pathway, particularly in the presence of activating mutations within this pathway.

We showed that YIF1B knockdown by shRNA in A‐375 and A‐875 melanoma cells significantly reduced cell motility and invasive capacity, as evidenced by wound healing and transwell assays. These findings are consistent with previous reports that YIF1B enhances metastatic potential by modulating the cytoskeleton, promoting extracellular matrix degradation, and upregulating epithelial–mesenchymal transition (EMT) markers [26–29]. qRT‐PCR and immunofluorescence confirmed efficient knockdown of YIF1B at both mRNA and protein levels. Collectively, our results support that YIF1B acts as a direct regulator of melanoma cell aggressiveness, in line with its described oncogenic roles in other cancer types such as urothelial carcinoma and colorectal adenocarcinoma [22–24].

Beyond its cell‐intrinsic effects, YIF1B contributes to an immunosuppressive TME. We observed that YIF1B‐overexpressing melanoma cells secrete increased levels of immunosuppressive cytokines (e.g., IL‐1A and IL‐12A) and upregulate immune checkpoint molecules (e.g., CD80 and CD28). Moreover, YIF1B facilitated the infiltration and activation of immunosuppressive cellular subsets, including CD8^+^ T cells, Th1 cells, and M0/M1 macrophages. Most importantly, we found that YIF1B significantly enhances the production and activity of IL‐6 in the TME. IL‐6 is a well‐known proinflammatory cytokine that, when chronically elevated, drives T cell exhaustion by upregulating inhibitory receptors (PD‐1 and Tim‐3) and impairing effector functions [16, 19]. Mechanistically, we demonstrated that YIF1B‐driven IL‐6 signaling leads to an exhausted gene signature in tumor‐infiltrating CD8^+^ T cells, characterized by reduced production of IFN‐*γ*, TNF‐*α*, and granzyme B. This YIF1B–IL‐6 axis, therefore, represents a critical mechanism by which melanomas directly disarm the major antitumor effector lymphocyte population.

A key finding of our study is that activating mutations in the IL‐6/JAK pathway (e.g., in IL6ST, JAK1, JAK2, or STAT3) frequently co‐occur with YIF1B overexpression in aggressive SKCM. Whole‐genome sequencing data from our cohorts revealed that patients harboring such mutations exhibited accelerated disease progression and the poorest survival outcomes. Functionally, these mutations potentiate downstream signaling, creating a positive feedback loop with YIF1B‐induced IL‐6 to amplify JAK/STAT activation. This synergistic interaction not only further enhances melanoma cell proliferation and metastasis but also deepens CD8^+^ T cell exhaustion, culminating in profound immunosuppression and unchecked tumor growth. Our observations align with recent studies showing that persistent IL‐6/JAK signaling promotes immune evasion in various cancers [16, 18, 19]. Thus, we propose that YIF1B overexpression and concurrent IL‐6/JAK pathway mutations jointly define an aggressive molecular subtype marked by intrinsic tumor aggressiveness and an immunologically “cold” microenvironment.

We acknowledge several limitations. First, we did not systematically investigate the post‐translational modifications of YIF1B or its regulatory elements. Second, the biological functions of YIF1B were validated only in vitro using melanoma cell lines; in vivo experiments in immunocompetent mouse models are needed to confirm its role in shaping the TME and driving T cell exhaustion. Third, the precise molecular mechanism by which YIF1B regulates IL‐6 expression (e.g., transcriptional, post‐transcriptional, or via trafficking of IL‐6R) requires further elucidation. Finally, the interaction between YIF1B and specific IL‐6/JAK pathway mutations should be explored in genetically engineered models. Future research should also examine whether YIF1B affects other JAK‐associated cytokine receptors and how its expression is regulated by tumor‐intrinsic or microenvironmental cues.

In summary, our study establishes YIF1B as a critical driver of melanoma progression that promotes both tumor cell aggressiveness and CD8^+^ T cell exhaustion through activation of the IL‐6/JAK pathway—particularly in tumors harboring activating mutations in this pathway. These findings highlight the YIF1B/IL‐6 axis as a potential prognostic biomarker and therapeutic target. Overcoming YIF1B‐mediated immune suppression may rejuvenate exhausted CD8^+^ T cells and improve the efficacy of immunotherapy in patients with SKCM.

## Author Contributions

Xiaohan Wang, Yangxu Ding, Wenbo Cai, Chaowei Liu, and Huijuan Shi drafted the manuscript. Huijuan Shi performed the literature search and collected the data. Huijuan Shi analyzed and visualized the data. Huijuan Shi helped with the final revision of this manuscript. Xiaohan Wang and Yangxu Ding contributed equally to this work.

## Funding

The Construction of Talent Platform Project of Ningxia (Innovation Team for Skin Disease Diagnosis and Treatment Technology and Drug Discovery and Development) (No. NXKJT2019012) funded this study to H.S.

## Disclosure

All authors reviewed and approved the final manuscript.

## Ethics Statement

The authors have nothing to report.

## Conflicts of Interest

The authors declare no conflicts of interest.

## Supporting Information

Additional supporting information can be found online in the Supporting Information section.

## Supporting information


**Supporting Information 1.** Figure S1: Enrichment analyses in cutaneous melanoma. (A) Pathway Cox regression analysis. (B) KEGG enrichment analysis. (C) Metabolic analysis.

**Supporting Information 2.** Figure S2: The expression of the YIF1B gene in cutaneous melanoma induces responsible immune changes. (A) The correlation changes between the YIF1B gene and immune expression. (B) The expression of immune changes in the high and low risk of the YIF1B gene. (C) The expression of immune examination inhibition points of the YIF1B gene.

**Supporting Information 3.** Figure S3: The expression of the YIF1B in cutaneous melanoma. (A) sh‐YIF1B mouse model. (B) The expression of the YIF1B in B16‐F10.

## Data Availability

The datasets analyzed in this study can be found in TCGA (https://portal.gdc.cancer.gov/) and GEO (https://www.ncbi.nlm.nih.gov/geo/).
