# Supplementary figures and images for: YIF1B Mutational Dysregulation Drives Cutaneous Melanoma Progression by Remodeling the TME
Source: Hum Mutat. 2026 May 30;2026:8907583. doi: 10.1155/humu/8907583 (PMC13239320; doi:10.1155/humu/8907583)

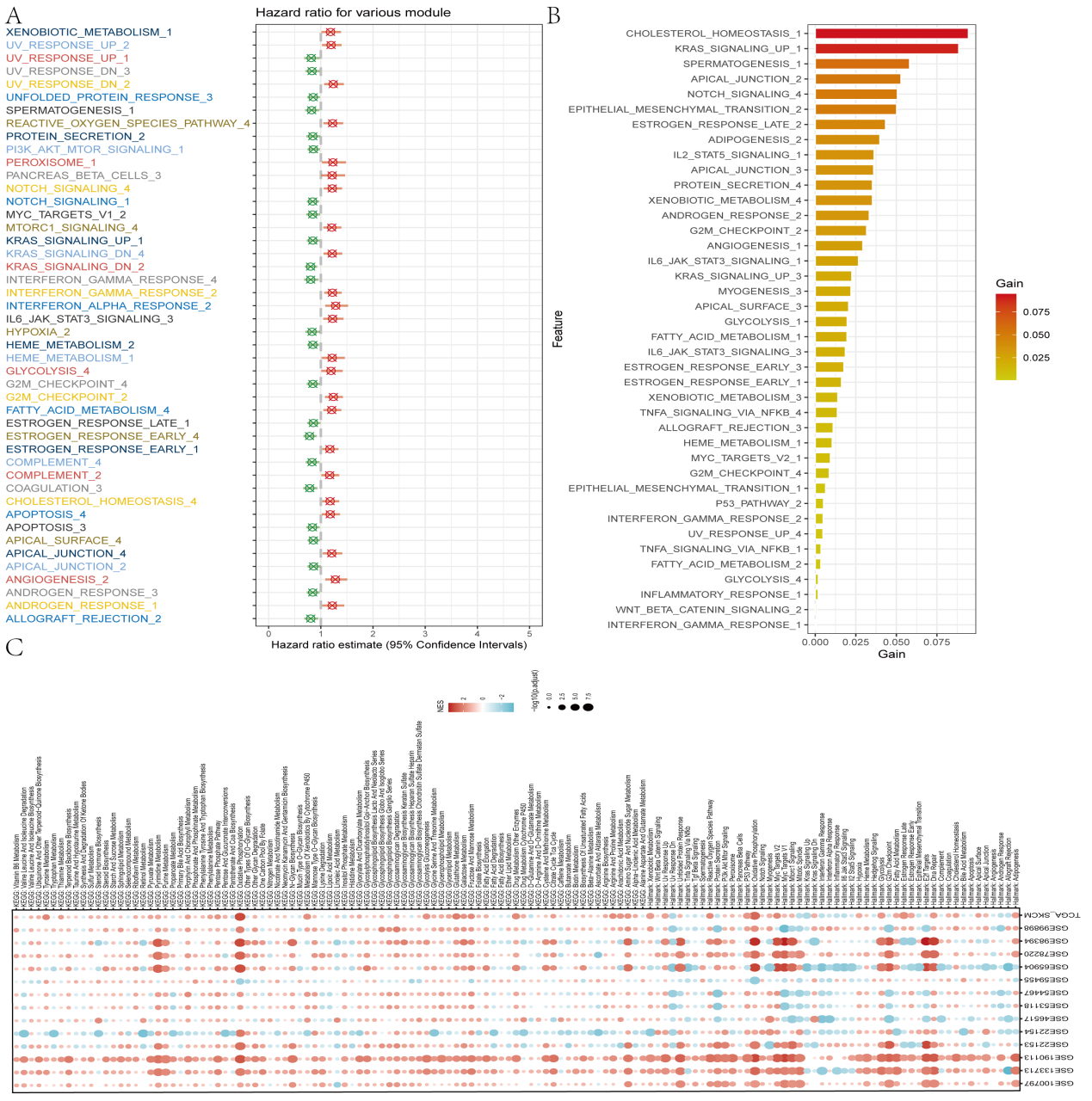

Supplement: Supplementary file 1 — Supporting Information 1. Figure S1: Enrichment analyses in cutaneous melanoma. (A) Pathway Cox regression analysis. (B) KEGG enrichment analysis. (C) Metabolic analysis. [file HUMU-2026-8907583-s003.tif]

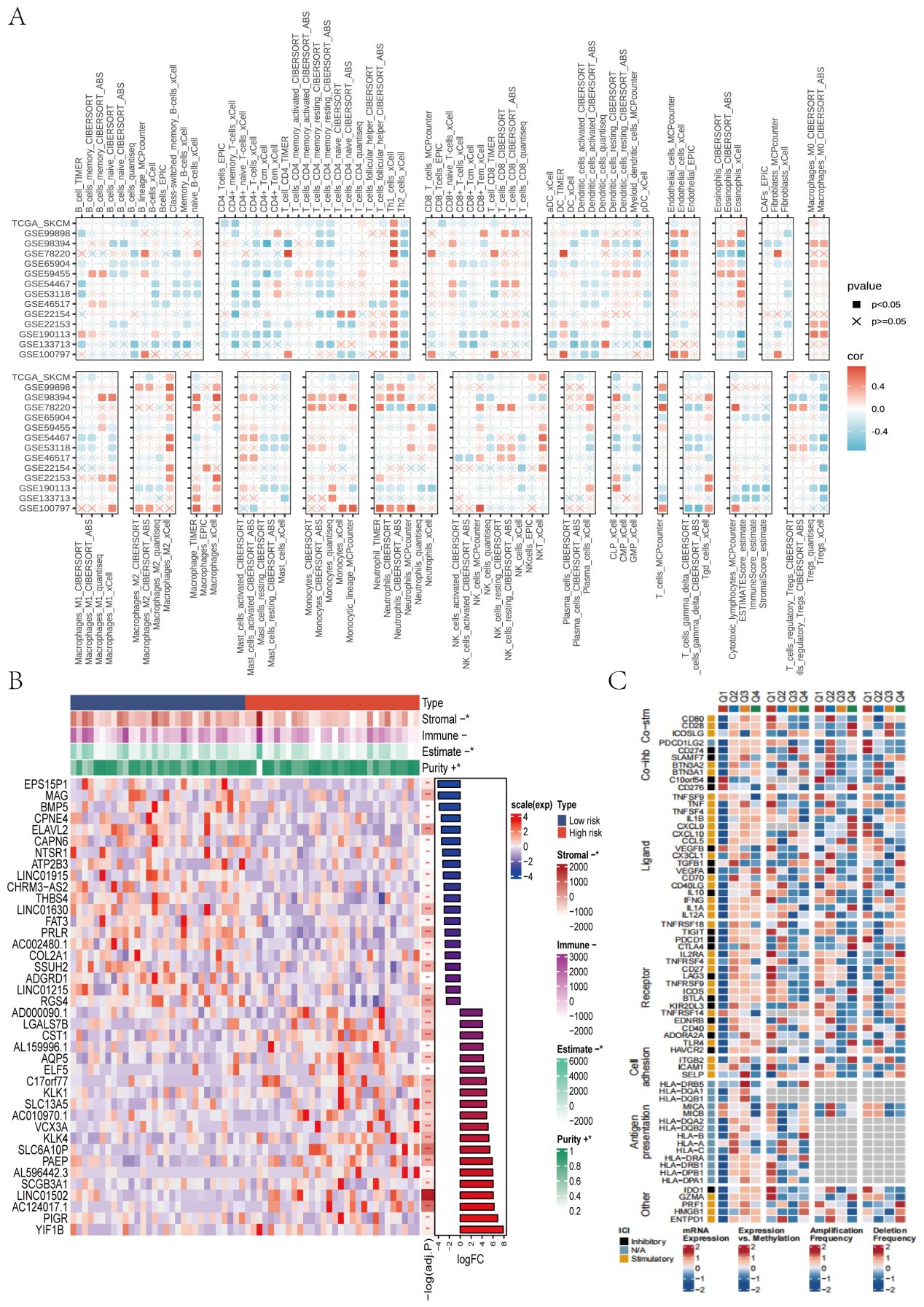

Supplement: Supplementary file 2 — Supporting Information 2. Figure S2: The expression of the YIF1B gene in cutaneous melanoma induces responsible immune changes. (A) The correlation changes between the YIF1B gene and immune expression. (B) The expression of immune changes in the high and low risk of the YIF1B gene. (C) The expression of immune examination inhibition points of the YIF1B gene. [file HUMU-2026-8907583-s002.tif]

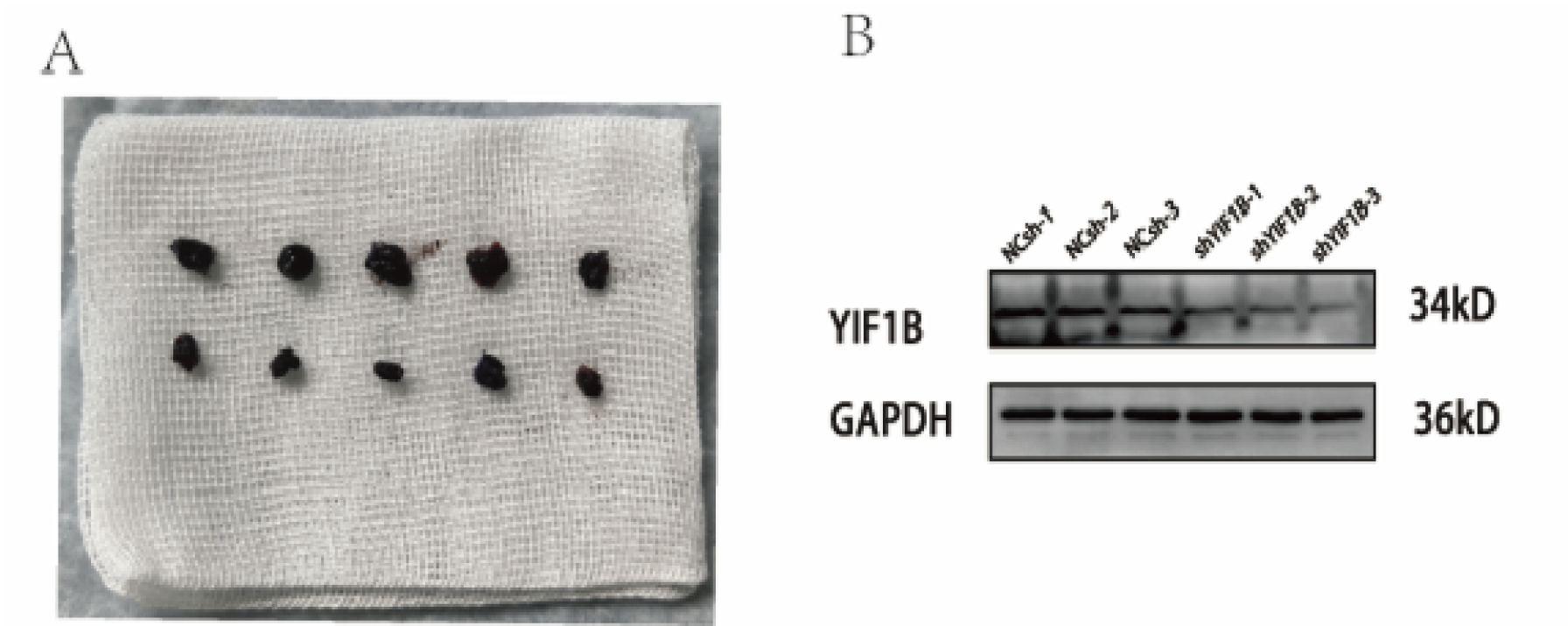

Supplement: Supplementary file 3 — Supporting Information 3. Figure S3: The expression of the YIF1B in cutaneous melanoma. (A) sh‐YIF1B mouse model. (B) The expression of the YIF1B in B16‐F10. [file HUMU-2026-8907583-s001.tif]
